# Childhood exposure to interpersonal and animal-directed violence: adversity profiles and adult suicidality

**DOI:** 10.3389/fpsyt.2026.1771930

**Published:** 2026-03-17

**Authors:** Shelby E. McDonald, Camie Tomlinson, Stacey Freedenthal, Charlotte L. Bright, Amelia Malone, Nicole Nicotera, Lori Kogan, Hannah Van Buiten, Jada Ford

**Affiliations:** 1 School of Social Work, Colorado State University, Fort Collins, CO, United States; 2 Kent School of Social Work and Family Science, University of Louisville, Louisville, KY, United States; 3 Graduate School of Social Work, University of Denver, Denver, CO, United States; 4 Clinical Sciences, College of Veterinary Medicine and Biomedical Sciences, Colorado State University, Fort Collins, CO, United States; 5 Independent Researcher, Kailua, HI, United States

**Keywords:** animal cruelty, childhood adversity, interpersonal violence, latent class analysis, psychological distress, suicide attempt, suicidal ideation, adverse childhood experiences

## Abstract

**Background:**

Exposure to animal-directed violence is an overlooked aspect of childhood adversity that frequently co-occurs with interpersonal violence and may indicate heightened developmental risk. The existence and implications of this co-occurrence for adult suicidality have not been investigated. To address this gap, we compared suicidality across empirically derived adversity profiles that varied in childhood exposure to interpersonal and animal-directed violence.

**Method:**

Data were drawn from 1,072 adults who completed the Pets, Attachment, and Mental Health Study through Prolific. Threat-based adverse childhood experiences were assessed using items from the WHO ACE-IQ, and childhood exposure to animal cruelty was assessed using items adapted from the Pet Treatment Survey. Latent class analyses identified three adversity profiles: Low Adversity, Exposure to Interpersonal Violence Only, and Exposure to Both Interpersonal Violence and Animal Cruelty. Suicidal ideation was measured using the Beck Scale for Suicide Ideation and the Suicidal Ideation Attributes Scale. Lifetime suicide attempt was assessed using a dichotomous item. Psychological distress, social support, and sociodemographic variables were included as covariates. Group differences were examined using the Bolck-Croon-Hagenaars method in Mplus.

**Results:**

Adults in the Interpersonal Violence and Animal Cruelty class reported higher intensity of recent suicidal ideation and greater odds of a lifetime suicide attempt compared with the Low Adversity and Interpersonal Violence Only classes. There were no differences between the Low Adversity and Interpersonal Violence Only classes on any suicidality outcome. Relative to heterosexual adults and cisgender men, sexual and gender minority adults were more likely to be represented in the Interpersonal Violence and Animal Cruelty class; similarly, relative to White participants, Black participants were more likely to be represented in this class.

**Conclusion:**

Co-occurring exposure to interpersonal violence and animal-directed violence in childhood is associated with elevated adult suicidality. These results identify animal cruelty exposure as a meaningful component of threat-based adversity and support its inclusion in trauma history interviews and suicide risk assessments.

## 1 Introduction

Suicide is one of the leading causes of death among adults in the United States and globally (1–3). Each year, as many as 720,000 people across the globe die as a result of suicide (3). Despite sustained investment in prevention and clinical response, rates of suicidal ideation and nonfatal attempts in the U.S. have increased in recent years (4–7). These dynamics underscore the importance of identifying developmental and contextual factors that shape long-term vulnerability to suicide in adulthood (8–10). Understanding how early experiences of adversity contribute to this vulnerability is essential for improving prevention, screening, and intervention strategies (11–14). Accordingly, this study examines whether empirically derived profiles of threat-based childhood adversity that vary in childhood exposure to animal cruelty are differentially associated with suicidal ideation in adulthood and lifetime history of suicide attempt.

### 1.1 ACEs and suicide risk

Adverse Childhood Experiences (ACEs) have been consistently linked to elevated suicide risk across the life span (11, 15–17). Blair et al.’s (15) systematic review and meta-analysis shows that in low- and middle-income countries, several ACEs—including emotional, verbal, psychological, physical, and sexual abuse; parental separation, divorce, or death; and bullying victimization—are associated with increased odds of suicidal ideation, planning, and attempts. Notably, participants with three or more ACEs showed the highest likelihood of suicidal outcomes. In a national U.S. longitudinal study, adults who had experienced physical abuse, sexual abuse, emotional abuse, parental incarceration, or a family history of suicidality were 1.4 to 2.7 times as likely to think about or attempt suicide in adulthood as those without these experiences (18).

Building on this evidence, research increasingly recognizes that childhood adversity is heterogeneous—different ACE types cluster into broader dimensions that carry distinct implications for development (19–21). One influential framework is the dimensional model of childhood adversity, which distinguishes deprivation (reduced cognitive and social inputs) from threat (exposure to harm or the threat of harm; 19-21). Threat-based adversities such as physical abuse, sexual abuse, and exposure to interpersonal or community violence have been associated with heightened stress reactivity, difficulties with emotion regulation, and maladaptive coping (19, 22, 23). These challenges associated with threat-based adversities can disrupt child development and persist into adulthood. They also align with mechanisms that precede and co-occur with suicidal thinking and behavior, including hopelessness, perceived burdensomeness, and social disconnection (10, 24, 25). On the other hand, it has been shown that social connectedness and a sense of belonging are among the protective factors that reduce suicide risk for adults (26–28).

Research consistently shows that ACEs tend to co-occur (29). Such clustering produces identifiable patterns of exposure that correspond to variation in adult mental-health outcomes (29–32). For example, Shin and colleagues (29) used latent class analysis (LCA) in a sample of young adults and found that poly−victimization classes showed markedly worse substance−use outcomes than low−adversity classes. Person−centered approaches such as LCA have been used to characterize this heterogeneity in patterns of ACE exposure by identifying subgroups defined by shared patterns of adversity exposure. This framework moves beyond cumulative scoring to reflect the qualitative structure of early experiences and provides a foundation for examining how distinct patterns of adversity are associated with psychological outcomes (33, 34). Importantly, these patterns are not evenly distributed across populations; sociodemographic factors such as gender, sexual orientation, race, and ethnicity have been linked to differential exposure to threat-based adversities (30, 32, 35, 36).

### 1.2 Exposure to animal cruelty as a form of threat-based adversity

Within this broader context, childhood exposure to animal cruelty represents an overlooked form of threat-based adversity that frequently co-occurs with interpersonal violence yet remains largely absent from multidimensional ACE frameworks (32, 37, 38). Despite being a well-documented correlate of family violence, animal cruelty has rarely been systematically assessed in research on childhood adversity (39, 40). U.S. Census data (41) suggest that nearly half of American households include companion animals, and families with children are more likely to have pets in the home. For many children, companion animals are integral to the household environment and serve as sources of comfort, attachment, and emotional regulation (42–44). Exposure to harm or threats toward these animals can therefore be deeply distressing and has been linked to greater risk for psychological difficulties in both childhood and adulthood relative to those who have not been exposed to animal cruelty (45–48). Empirical studies indicate that exposure to animal cruelty frequently overlaps with emotional, physical, and sexual abuse, as well as children’s exposure to domestic violence (38, 40, 49). These experiences expose children to threat, loss, and moral injury, processes that can disrupt safety, empathy, and relational security and align with established mechanisms of threat-based adversity (50–52). Including exposure to animal cruelty within threat-based ACE models therefore offers a more comprehensive representation of violence exposure and its developmental and psychological correlates than standard ACEs alone.

McDonald et al. (32) applied LCA to identify empirically derived subgroups of adults based on their histories of childhood physical, sexual, and emotional abuse and exposure to domestic violence and animal cruelty. The resulting three-class model included a low-adversity group, a class characterized by exposure to interpersonal violence without animal cruelty, and a class characterized by exposure to both interpersonal violence and animal cruelty. Adults in the latter class reported the highest levels of depression, anxiety, and stress, indicating that co-occurring exposure to human- and animal-directed violence is associated with elevated psychological distress. Although prior research has linked adversity profiles to suicidality, no study, to our knowledge, has examined these associations in models that also account for childhood exposure to animal cruelty.

### 1.3 Current study

Building on the latent classes established by McDonald et al. (32), the present study tests whether adults with distinct adversity profiles (i.e., Low Adversity, Exposure to Interpersonal Violence Only, and Exposure to Interpersonal Violence and Animal Cruelty) differ in their likelihood of reporting recent suicidal ideation and a lifetime suicide attempt, while adjusting for overall levels of psychological distress, perceived social support, and sociodemographic factors. We hypothesized that adults in the Exposure to Interpersonal Violence and Animal Cruelty subgroup would report greater severity and intensity of suicidal ideation and higher odds of a lifetime suicide attempt compared with both the Low Adversity and Interpersonal Violence Only subgroups.

## 2 Method

### 2.1 Participants and procedure

All participants (*N* = 1,072) were adults aged 18 years and older who completed an anonymous online survey in English assessing childhood experiences, relationships with animals, and adult psychological functioning. Participants were recruited through Prolific, an online research platform, as part of the Pets, Attachment, and Mental Health Study, a national online survey approved by the Colorado State University Institutional Review Board. The sample had a mean age of 46.36 years (SD = 16.16) and represented multiple gender modalities, with most identifying as cisgender women (48.2%) or cisgender men (46.1%), and the remainder identifying as a minoritized gender (5.7%). Participants identified as White/non-Hispanic (62.6%), Hispanic (12.1%), Black (12.1%), and other racial/ethnic identities (13.2%). Additional sample characteristics, including relationship status, sexual orientation, and pet-ownership history, are provided in McDonald et al. (32).

### 2.2 Measures

#### 2.2.1 ACE classes

Threat-based ACEs (emotional, physical, and sexual abuse, and exposure to domestic violence) were assessed using items from the World Health Organization’s *Adverse Childhood Experiences–International Questionnaire* (ACE-IQ; 53, 54), as described in detail in McDonald et al. (32). Participants rated the frequency of each experience on a five-point scale from *never* to *always*. Items were dichotomized to indicate any exposure (1 = ever, 0 = never).

Childhood exposure to animal cruelty was assessed with two items adapted from the *Pet Treatment Survey* (55), which asked whether participants had ever seen or heard a parent, guardian, sibling, or other household member intentionally harm a pet (i.e., *Did you see or hear a parent, guardian, or other adult household member hurt a pet on purpose? Did you see or hear a sibling or another child in your household hurt a pet on purpose*)*?*. Responses were rated on a five-point scale from *never* to *always* and dichotomized to indicate any exposure. These animal-cruelty items, along with the four threat-based ACE domains, served as indicators within the previously identified latent classes. Additional details on dichotomization and psychometric properties are provided in McDonald et al. (32).

Latent classes representing patterns of threat-based adversity were identified in McDonald et al. (32). Three classes emerged (1): Low Adversity (2), Exposure to Interpersonal Violence Only, and (3) Exposure to Interpersonal Violence and Animal Cruelty. The present study uses this 3-class categorization of ACEs as the independent variable to examine associations with suicidality.

#### 2.2.2 Suicidality

Suicidality was assessed using multiple indicators capturing related but distinct aspects of suicidal thoughts and behavior. Specifically, we assessed severity of recent suicidal ideation, lifetime history of suicide attempt, and intensity of suicidal ideation. In this study, severity of suicidal ideation reflects the degree of suicidal desire, intent, and planning, whereas intensity reflects the subjective experience of suicidal thoughts, including their frequency, controllability, associated distress, and interference with functioning (56–58).

Severity of past-week suicidal ideation was assessed using 19 items from the Beck Scale for Suicide Ideation (BSS; 55). The BSS includes 21 items evaluating past-week suicidal thoughts, planning, and intent, each rated on a three-point scale (0–2). For this study, the phrase “commit suicide” was replaced with “die by suicide” per contemporary guidance (e.g., Nielsen, Padmanathan, & Knipe, 2016 [56]). The first five items were treated as screening items. Participants who did not endorse past-week suicidality on the first five screening items (i.e., they selected the lowest-risk options: “I have a moderate to strong wish to live,” “I have no wish to die,” “My reasons for living outweigh my reasons for dying,” “I have no desire to kill myself,” and “I would try to save my life if I found myself in a life-threatening situation”) did not see the remaining 14 items. Survey logic was used to administer the follow-up items only to participants who endorsed any level of suicidal desire or ambivalence on the initial screening items. Total suicidal ideation scores were calculated by summing the items answered. Participants who did not endorse any of the screening items had a score of 0, and the total scores for participants who did endorse at least one screening item were based on the sum of their responses to all 19 items with higher scores indicating more severe ideation. Internal consistency for the BSS total score was good (ω = .86).

All participants were shown BSS item 20, which states: *“Select the one statement that best describes your history of suicide attempts across your lifetime.”* The three response options were: *0 = I have never attempted suicide; 1 = I have attempted suicide once; 2 = I have attempted suicide two or more times.* Item 20 served as our measure of lifetime suicide attempts. Responses were recoded into a dichotomous variable indicating whether participants had never (=0) or had ever (=1) attempted suicide (i.e., “once” or “two or more times”). BSS item 21, which asked about intent to die during a suicide attempt, was not administered in this study because the analytic focus was on lifetime history of suicide attempt rather than characteristics of attempt intent or lethality, and to minimize participant burden in an online survey context.

Intensity (i.e., subjective experience) of suicidal ideation was assessed using the Suicidal Ideation Attributes Scale (SIDAS) (58). The SIDAS consists of five items assessing how often the person thought of suicide in the past month, how much control the person had over their thoughts, how tormented they felt by them, how much their suicidal thoughts interfered with functioning, and how close the person came to attempting suicide. Items are rated on 11-point Likert-type scale ranging from 0 to 10, with higher values indicating more intense ideation in the past month. Individuals who indicated in the first item that they had no suicidal thoughts in the previous month skipped the remaining items, which were scored 0. Total scores were calculated by summing all items, yielding a range from 0 to 46 (out of a maximum score of 50). Internal consistency for the SIDAS total score was good (ω = .83).

#### 2.2.3 Psychological distress

Overall psychological distress was assessed using the Depression, Anxiety, and Stress Scale–21 (DASS-21; 59) and included as a covariate to examine whether associations between adversity profiles and suicidality persisted beyond current overall symptom burden. The DASS-21 is a 21-item self-report measure designed to capture symptoms of depression, anxiety, and stress in the past week as a single composite indicator of emotional distress. Participants rated each item on a four-point Likert-type scale ranging from *Never* to *Almost Always*. Total scores were calculated by summing all item responses and multiplying the result by two to maintain comparability with the original 42-item version, which facilitates interpretability of descriptive values and consistency with established severity thresholds. This linear rescaling does not affect model estimation or inference. Higher total scores indicate greater overall psychological distress. The average DASS score in our sample was 27.03 (*SD =* 26.15), and internal consistency for the DASS-21 total score in the present study was excellent (ω = .98).

#### 2.2.4 Social support

Perceived social support was included as a covariate and measured using the Multidimensional Scale of Perceived Social Support (MSPSS; 60). The MSPSS includes 12 items assessing support from family, friends, and significant others. Each item is rated on a seven-point Likert-type scale ranging from *Very Strongly Disagree* to *Very Strongly Agree*. A total perceived support score was calculated by averaging all items, with higher scores reflecting greater perceived support. The mean perceived social support score in our sample was 5.23 (*SD* = 1.19). Internal consistency of the MSPSS total score was excellent (ω = .95).

#### 2.2.5 Sociodemographic variables

Sociodemographic covariates included age, gender modality, sexual orientation, race/ethnicity, relationship status, childhood pet ownership, and current pet ownership. Age was entered as a continuous variable. Gender identity and modality were captured in a single variable dummy-coded into cisgender man (reference group), cisgender woman, and gender minority. Sexual orientation was coded as 0 = heterosexual and 1 = sexual minority. Relationship status was dichotomized to indicate whether participants were single/not in a relationship (=0, reference group) or in a relationship (=1). Pet ownership variables were also dichotomized such that those who did not live with a pet in childhood (=0) or currently (=0) were used as the reference group in comparison to those who had lived with a pet in childhood (=1) or currently lived with a pet (=1). Race and ethnicity were represented by three dummy variables comparing Hispanic, Black/non-Hispanic, and other racial or ethnic identities with White/non-Hispanic as the reference category; the “other” group included participants who identified as Asian, multiracial, Middle Eastern, Native American/Indigenous, or Native Hawaiian/Pacific Islander. These codings follow those used in McDonald et al. (32) and reflect established demographic distinctions associated with both adversity exposure and suicide risk.

### 2.3 Analytic plan

SPSS version 30 (61) was used for data cleaning and assumption checks; main analyses were conducted in Mplus version 8.10. Multicollinearity checks for each of the dependent variables were satisfied based on all VIF values < 10. Missing data were handled using full information maximum likelihood (FIML) estimation, which uses all available observed data to estimate model parameters without imputing missing values, under the assumption that data were missing at random. FIML incorporated missingness on outcome variables and covariates included in the regression models. However, we chose to remove participants missing on *all* ACE indicators (*n* = 11, <1% missing) and *any* covariate (*n* = 75; at least one participant was missing on each covariate) so that all analyses were conducted with the same sample (i.e., participants not included in the LCA were not later included in the regression analyses). Descriptive statistics and bivariate correlations for the dependent variables were examined to characterize the sample (see **Table 1**). We used LCA to identify underlying subgroups based on similar patterns of endorsing the five ACEs indicators (i.e., exposure to sexual abuse, emotional abuse, physical abuse, domestic violence, and animal cruelty). A full description of the latent class enumeration process can be found in McDonald et al. (32).

**Table 1:** Correlations and descriptives of the suicidality variables (N = 1072).

Suicidality variables	1	2	3	*M*	*SD*	#	%
SIDAS total score	–			6.68	10.04	396	36.9
BSS total score	0.69***	–		2.32	5.25	261	24.3
Ever attempted suicide	0.36***	0.35***	–	0.19	0.39	207	19.3

SIDAS, Suicidal Ideation Attributes Scale, BSS, Beck Scale for Suicide Ideation. Ever attempted suicide was a dichotomous variable, such that 0 = no, 1 = yes. The # and % in the table reflect the number of participants who endorsed thoughts of suicide in the past month (SIDAS), any of the first 5 screening items on the BSS, or having ever attempted suicide, respectively.

To examine latent subgroup differences in covariates and suicidality variables, we used the Bolck-Croon-Hagenaars [BCH; (62)] approach, which accounts for individual classification error through the creation of weights and prevents shifts in the latent classes in subsequent analyses. To test for latent subgroup differences in covariates, we regressed the latent class variable on age, gender modality, sexual orientation, race/ethnicity, and childhood pet ownership. These logistic regression results were provided in Mplus in log odds format, which were then converted into odds ratios by exponentiating the coefficients. Next, we examined whether there were significant differences in suicidality across latent subgroups, adjusting for covariates (age, gender modality, sexual orientation, relationship status, social support, race/ethnicity, and current pet ownership). Severity of suicidal ideation (BSS total scores) and intensity of suicidal ideation (SIDAS total scores) were modeled as continuous outcomes using linear regression, and lifetime suicide attempt (ever vs. never) was modeled using logistic regression. Wald tests were used to determine whether each association was statistically significant, and, if so, pairwise differences were explored using the Mplus model constraint function.

## 3 Results

### 3.1 Latent class enumeration

Three threat-based adversity profiles identified in our prior work (32) were used in the present analyses: Low Adversity (29.6% of sample), Exposure to Interpersonal Violence Only (34.8%), and Exposure to Interpersonal Violence and Animal Cruelty (35.6%). Complete model fit statistics and class probabilities are reported in the earlier publication.

### 3.2 Sociodemographic differences in subgroup membership

There were significant differences in subgroup membership based on age (X^2^(2) = 8.00, *p* = .018), gender modality (X^2^(4) = 12.81, *p* = .012), sexual orientation (X^2^(2) = 14.45, *p* = .001) and race/ethnicity (X^2^(6) = 22.11, *p* = .001). Descriptive statistics for all study variables are provided within each latent class in **Table 2** and specific differences in odds of subgroup membership based on sociodemographic characteristics are provided in **Table 3**. In summary, older participants had lower odds of being in the Interpersonal Violence and Animal Cruelty subgroup versus the Low Adversity subgroup in comparison to younger participants, and individuals with a minoritized gender modality and/or sexual orientation, and Black individuals had higher odds of being in the Interpersonal Violence and Animal Cruelty subgroup versus the Low Adversity subgroup in comparison to cisgender men, heterosexual individuals, and White individuals, respectively. There were no significant differences in subgroup membership across childhood pet ownership (X^2^(2) = 3.47, *p* = .177).

**Table 2:** Descriptive statistics of covariates and suicidality variables across the 3-class model and in the full sample.

Variable	Class 1 *n* = 317	Class 2 *n* = 373	Class 3 *n* = 382	Full Sample (*N* = 1072)
Age	48.73 (15.68)	46.77 (15.94)	43.99 (16.47)	46.36 (16.16)
Sexual orientation				
Heterosexual*	287 (90.5%)	325 (87.1%)	282 (73.8%)	894 (83.40%)
Sexual minority	30 (9.5%)	48 (12.9%)	100 (26.2%)	178 (16.60%)
Gender modality				
Cisgender woman	151 (47.6%)	178 (47.7%)	188 (49.2%)	517 (48.23%)
Cisgender man*	161 (50.8%)	183 (49.1%)	150 (39.3%)	494 (46.08%)
Gender minority	5 (1.6%)	12 (3.2%)	44 (11.5%)	61 (5.69%)
Childhood pet				
No*	44 (13.9%)	42 (11.3%)	65 (17.0%)	151 (14.09%)
Yes	273 (86.1%)	331 (88.7%)	317 (83.0%)	921 (85.91%)
Race/ethnicity				
Hispanic	36 (11.4%)	42 (11.3%)	52 (13.6%)	130 (12.13%)
White/non-Hispanic*	211 (66.6%)	231 (61.9%)	229 (59.9%)	671 (62.59%)
Black/non-Hispanic	26 (8.2%)	36 (9.7%)	68 (17.8%)	130 (12.13%)
Other race	44 (13.9%)	64 (17.2%)	33 (8.6%)	141 (13.15%)
Current pet ownership				
No	52 (16.4%)	66 (17.7%)	41 (10.7%)	159 (14.83%)
Yes	265 (83.6%)	307 (82.3%)	341 (89.3%)	913 (85.17%)
Relationship status				
Single	130 (41.0%)	173 (46.4%)	138 (36.1%)	441 (41.14%)
Partnered / married	187 (59.0%)	200 (53.6%)	244 (63.9%)	631 (58.86%)
Psychological distress	13.36 (16.60)	22.73 (21.07)	42.58 (29.05)	27.03 (26.15)
Perceived social support	5.55 (1.04)	5.23 (1.20)	4.97 (1.24)	5.23 (1.19)
SIDAS total score	2.91 (6.10)	5.10 (8.19)	11.34 (12.31)	6.68 (10.05)
BSS total score	0.86 (2.88)	1.91 (4.50)	3.94 (6.81)	2.32 (5.25)
Ever attempted suicide				
No	286 (90.2%)	317 (85.0%)	262 (68.6%)	865 (80.69%)
Yes	31 (9.8%)	56 (15.0%)	120 (31.4%)	207 (19.31%)

The means and standard deviations for continuous variables and proportions for categorical variables were computed by latent class using participants’ most likely class assignments from Mplus to summarize covariate distributions within each latent class. Class 1 = Low adversity, Class 2 = Interpersonal violence only, Class 3 = Interpersonal violence and animal cruelty. * indicate reference group in covariate analysis.

**Table 3:** Logistic regression model results of the association between covariates and latent class membership.

Covariate	Class 2 vs. Class 1			Class 3 vs. Class 1		
	OR	95% CI LL	95% CI UL	OR	95% CI LL	95% CI UL
Age (continuous): X^2^(2) = 8.00, *p* = .018	0.99	0.98	1.01	**0.98**	**0.92**	**0.99**
Gender modality (ref = cisgender man): X^2^(4) = 12.81, *p* = .012						
Cisgender woman	1.00	0.69	1.45	1.41	0.97	2.05
Gender minority	1.25	0.24	6.51	**5.35**	**1.65**	**17.36**
Sexual orientation (ref = heterosexual): X^2^(2) = 14.45, *p* = .001						
Sexual minority	1.15	0.60	2.20	**2.67**	**1.53**	**4.65**
Race/ethnicity (ref = white): X^2^(6) = 22.11, *p* = .001						
Hispanic	0.96	0.53	1.76	1.17	0.68	2.03
Black/non-Hispanic	1.13	0.56	2.26	**2.57**	**1.45**	**4.54**
Other race	1.43	0.86	2.40	0.50	0.25	1.01
Childhood pet (ref = no): X^2^(2) = 3.47, *p* = .177						
Yes	1.45	0.82	2.55	0.80	0.48	1.33

Bolded values indicate statistically significant differences in odds of latent class membership. Class 1 = Low adversity (=reference group), Class 2 = Interpersonal violence only, Class 3 = Interpersonal violence and animal cruelty.

### 3.3 Differences in suicidality outcomes across subgroups

**Figure 1:**
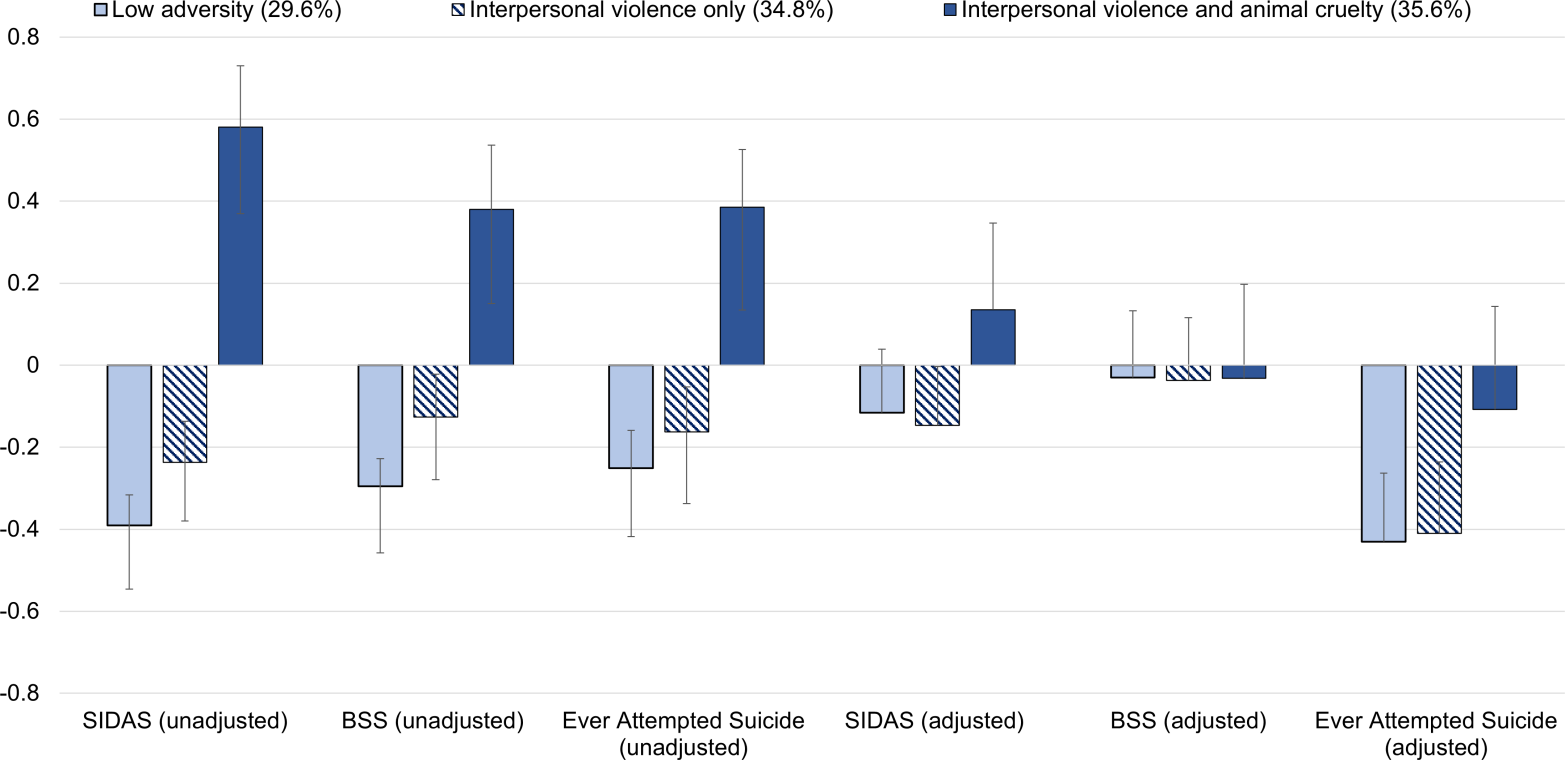
Standardized mean scores of suicidality variables for each subgroup with (adjusted) and without (unadjusted) covariates (N = 1,072). SIDAS, Suicidal Ideation Attributes Scale; BSS, Beck Scale for Suicide Ideation. Y-axis values represent standard deviations from the sample mean. Error bars represent 95% confidence intervals. Due to the dichotomous nature of the “ever attempted suicide variable,” the figure represents the standardized proportion of each class that endorsed a suicide attempt.

We present unadjusted and adjusted subgroup differences in suicidality in **Table 4**. By including covariates in the model, the significant differences in intensity of suicidal thoughts between the Low Adversity and Interpersonal Violence Only subgroups, as well as the significant differences in suicidal ideation severity scores, became non-significant. We present the adjusted results in more detail below. We found evidence of significant mean differences in intensity of suicidal thoughts (X^2^(2) = 6.71, *p* = .035) and significant differences in the proportion of participants who reported having ever attempted suicide (X^2^(2) = 7.98, *p* = .019) across classes, but no significant mean differences in suicidal ideation severity scores as measured by the BSS (X^2^(2) = 0.01, *p* = .994). The standardized means of each outcome across each subgroup are displayed in **Figure 1**. Differences in suicidal ideation intensity and lifetime suicide attempt were observed for the subgroup characterized by co-occurring exposure to interpersonal violence and animal cruelty. Specifically, the Interpersonal Violence and Animal Cruelty subgroup reported greater intensity of suicidal thoughts (i.e., SIDAS scores) compared with the Low Adversity (*d* = 0.25, *p* = .015) and Interpersonal Violence Only subgroups (*d* = 0.28, *p* = .011). The Interpersonal Violence and Animal Cruelty subgroup was more likely to have ever attempted suicide than the Low Adversity (*d* = 0.32, *p* = .005) and Interpersonal Violence Only subgroups (*d* = 0.30, *p* = .018). No differences were observed between the Low Adversity and Interpersonal Violence Only subgroups on suicidal ideation severity, suicidal ideation intensity, or lifetime suicide attempt.

**Table 4:** Unadjusted and adjusted subgroup differences in suicidality.

Class comparison	Unadjusted models						Adjusted models					
	SIDAS		BSS		Suicide Attempt		SIDAS		BSS		Suicide Attempt	
	*d*	*p*-value	*d*	*p*-value	*d*	*p*-value	*d*	*p*-value	*d*	*p*-value	*d*	*p*-value
Overall X^2^	**135.80**	**<.001**	**66.87**	**<.001**	**55.83**	**<.001**	**6.71**	**.035**	0.01	.99	**7.98**	**.019**
Class 1 vs. 2	**-0.16**	**.023**	**-0.17**	**.013**	-0.09	.26	0.03	.57	0.01	.91	-0.02	.79
Class 1 vs. 3	**-0.97**	**<.001**	**-0.68**	**<.001**	**-0.64**	**<.001**	**-0.25**	**.015**	0.001	.99	**-0.32**	**.005**
Class 2 vs. 3	**-0.82**	**<.001**	**-0.51**	**<.001**	**-0.55**	**<.001**	**-0.28**	**.011**	-0.01	.97	**-0.30**	**.018**

Overall Wald chi-square test results are presented in the first row, each with 2 degrees of freedom. The other rows reflect the Cohen’s *d* effect sizes of the standardized mean differences in each suicidality score across each subgroup comparison. The suicide attempt variable was dichotomous, so the results should be interpreted as the difference in proportions of those who endorsed ever having attempted suicide. Class 1 = Low Adversity, Class 2 = Interpersonal Violence Only, and Class 3 = Interpersonal Violence and Animal Cruelty. Bolded values indicate statistically significant subgroup differences in suicidality.

## 4 Discussion

Using latent adversity profiles (Low Adversity, Exposure to Interpersonal Violence Only, and Exposure to Interpersonal Violence and Animal Cruelty) previously identified within this sample (32), the current study tested whether distinct patterns of threat-based childhood adversity were differentially associated with adult suicidality when accounting for psychological distress, perceived social support, and sociodemographic factors. Two key patterns emerged: 1) individuals in the Interpersonal Violence and Animal Cruelty subgroup reported more intense suicidal thoughts, as assessed by SIDAS scores, than those in both the Low Adversity and Interpersonal Violence Only subgroups, and 2) individuals in the Interpersonal Violence and Animal Cruelty subgroup were also more likely to report a lifetime suicide attempt than either comparison group. In contrast, adults in the Interpersonal Violence Only subgroup did not differ from those in the Low Adversity subgroup on intensity (i.e., measured by SIDAS) of suicidal thoughts or on the likelihood of having ever attempted suicide once covariates were included.

### 4.1 Exposure to animal-directed violence and potential mechanisms of risk for suicidality

The present study’s findings suggest that the co-occurrence of animal-directed violence and interpersonal violence marks a particularly harmful developmental context. In our prior latent class work with this same sample (32), adults in this subgroup reported the highest levels of depression, anxiety, and stress. The current findings extend that pattern by showing elevated intensity of suicidal thoughts and higher likelihood of suicide attempts, even after adjusting for psychological distress. These results suggest that exposure to animal cruelty does not simply add another adverse event but may compound the effects of interpersonal violence in ways that magnify developmental and psychological risk.

Although this study did not test the developmental and psychological processes linking co-occurring human- and animal-directed violence exposure to suicidality, several theoretically informed pathways may help explain the heightened vulnerability observed in this subgroup. One pathway involves attachment and loss. Companion animals often function as attachment figures (63), particularly in homes where human relationships are inconsistent, frightening, or harmful (49, 64). When caregivers harm or threaten to harm a pet, children may experience a dual betrayal: the loss or endangerment of a cherished animal alongside the realization that adults responsible for their protection are sources of danger. Such experiences may erode foundational assumptions about safety, care, and relational reliability, contributing to hopelessness and social disconnection (65).

A second theoretically-grounded pathway involves moral injury and disruptions to children’s relational worlds. In their seminal paper on moral injury, Litz et al. (66) state that moral injury results from “perpetrating, failing to prevent, bearing witness to, or learning about acts that transgress deeply held moral beliefs and expectations” (p. 700). Witnessing animal cruelty perpetrated by trusted adults could violate a child’s moral beliefs about right, wrong, and responsibility (65). Children may experience guilt or shame for being unable to protect a pet, fear the consequences of intervening, or feel responsible for the animal’s suffering (49). The extent to which such experiences constitute moral injury is likely to vary depending on contextual factors, including whether animal-directed violence was normalized within the household and the child’s perceived capacity to intervene or prevent harm.

Moral injury can lead to self-directed emotions such as guilt, shame, and self-blame (66), emotions that in turn can increase vulnerability to suicidal thoughts and behaviors (67). Many children engage in protective behaviors—hiding animals, anticipating violence, intervening during episodes of harm to animals—and when those efforts fail, the resulting shame, responsibility, and loss of control may further erode coping resources (49, 68). Although most research on moral injury pertains to specific occupations (e.g., veterinary medicine, military, nursing), research supports that moral injury is linked to suicidal thoughts and behavior, even after accounting for posttraumatic stress and other mental illness (69–71).

Finally, chronic exposure to threatening environments can disrupt the development of emotion regulation and coping skills (72). Threat-based adversities are linked to heightened stress reactivity, difficulties modulating intense affect, and increased reliance on maladaptive coping strategies such as self-harm and substance use (73). These disruptions may serve as proximal mechanisms through which exposure to both interpersonal violence and animal cruelty is linked to increased risk for adult suicidality or lifetime history of suicide attempt, above and beyond global psychological distress. Future research should explicitly test these pathways.

### 4.2 Interpreting suicidality risk across adversity profiles

It is notable that, after adjustment for covariates, adults in the Interpersonal Violence Only subgroup did not differ from those in the Low Adversity subgroup on either measure of suicidal ideation or on history of suicide attempt. This finding contrasts with prior research linking exposure to interpersonal violence in childhood with elevated risk for suicidality [e.g., Castellvi et al., 2017 (74)]. Several explanations for this finding are possible. First, the absence of statistically significant group differences may reflect differences in severity, chronicity, or complexity of interpersonal violence between the Interpersonal Violence Only and Interpersonal Violence and Animal Cruelty classes. Because our indicators were dichotomized (any vs. no exposure), individuals who experienced relatively infrequent or less severe interpersonal violence could be grouped with those who experienced severe or chronic abuse. If animal cruelty tends to co-occur with the most extreme or coercively controlling forms of family violence, then the Exposure to Interpersonal Violence and Animal Cruelty subgroup may disproportionately capture cases at the highest end of the severity spectrum, where effects on suicidality are strongest (40, 75). Additionally, one specific ACE that had higher endorsement in the Interpersonal Violence and Animal Cruelty class was sexual abuse. This could potentially drive some portion of the association between class membership and suicidality, given that sexual abuse specifically has been linked to suicide attempts (11, 76, 77).

Consistent with prior research, higher perceived social support was associated with lower suicidality, suggesting that protective factors may help buffer suicide risk among some individuals exposed to interpersonal violence but may be insufficient in the context of more pervasive and co-occurring forms of threat-based adversity. Finally, unmeasured protective factors may be more prevalent in the Interpersonal Violence Only subgroup than in the Interpersonal Violence and Animal Cruelty subgroup, such as stable relationships with non-abusive caregivers, teachers, or peers, or early access to mental health services. It is also possible that unmeasured protective factors, such as the presence of a non-abused companion animal, were more prevalent in the Interpersonal Violence Only subgroup and contributed to lower suicidality in this group (48). These possibilities highlight the need for more nuanced measurement of both adversity and resilience in future work.

### 4.3 Measurement considerations

It is notable that adversity profiles were associated with intensity of suicidal ideation as measured by the SIDAS, but not with severity of ideation as measured by the BSS. This pattern highlights the importance of distinguishing between different dimensions of suicidal ideation when interpreting associations with childhood adversity. Several measurement-related factors may explain this discrepancy. First, the SIDAS measures past-month suicidal thoughts, and the BSS covers only the past week. The SIDAS therefore may have measured a larger and more diverse subsample of people who had recently thought of suicide. Second, the SIDAS measures the intensity of individuals’ subjective experiences of suicidal thoughts - frequency, controllability, distress caused by suicidal ideation, interference with functioning, and coming close to an attempt. In contrast, the BSS was developed as a scale to measure the severity of suicidal intent (78), though it also assesses suicidal desire and planning. Third, responses for the SIDAS are measured on a 10-point scale, and the BSS has only 3 response options per item. Thus, the SIDAS is more sensitive to a wider range of attributes of suicidal ideation across a longer period of time, which might account for the differential results. Although both measures demonstrated adequate internal consistency, our findings underscore that conclusions about associations between childhood adversity and suicidality can depend on how suicidal ideation is operationalized and measured.

### 4.4 Sociodemographic patterns and structural inequities

Differences in adversity profile membership and suicidality across sociodemographic groups observed in this study align with prior research documenting unequal exposure to violence and adversity across structurally marginalized populations. In comparison to cisgender men and heterosexual adults, gender minority and sexual minority adults, respectively, were less likely to be classified in the Low Adversity subgroup and more likely to fall into the Interpersonal Violence and Animal Cruelty subgroup; this class was most at risk for more intense suicidal thoughts and higher odds of a prior suicide attempt. These findings align with research documenting elevated rates of ACEs [e.g., Tran, Henkhaus, & Gonzales, 2022 (79)] and suicidality [e.g., Chum et al., 2023 (80)] among sexual and gender minority populations. Black participants were less likely than White participants to be classified in the Low Adversity subgroup and more likely to be in the Interpersonal Violence and Animal Cruelty subgroup; as previously discussed in McDonald et al. (32), this pattern likely reflects downstream effects of structural racism, including differential exposure to community and family violence, economic hardship, and constrained access to protection and supportive services (81). Overall, these patterns reinforce the need for intersectional approaches that consider how race, ethnicity, gender, and sexual orientation—together with patterns of childhood adversity—shape suicide risk (82).

### 4.5 Rates of suicidality

Study participants reported higher rates of suicidal ideation and suicide attempt than those observed in U.S. population-based studies. Approximately 37% endorsed suicidal ideation in the past month and 24% reported suicidal thoughts in the past week, compared with 5% of respondents in the National Survey on Drug Use and Health who reported serious suicidal ideation in the prior year (83). Similarly, 19% of participants reported a lifetime suicide attempt, compared with 5.4% in the National Epidemiologic Study of Alcohol and Related Conditions III (NESARC-III) (84). These comparisons are intended to provide contextual benchmarks rather than direct prevalence equivalence, given differences in measurement instruments, reference periods, and sampling frames. The elevated rates observed here likely reflect self-selection into an online mental health study and the explicit mention of suicidality in the consent process; accordingly, this sample should be understood as a relatively high-risk group rather than representative of the general U.S. population.

### 4.6 Clinical and public health implications

From a clinical and public health perspective, the current findings suggest that childhood exposure to animal cruelty when combined with interpersonal violence should be treated as a marker for increased suicide risk. Routine assessment of trauma and ACEs in mental health and primary care settings rarely includes questions about harm or threats to pets. Incorporating brief, behaviorally specific questions about animal-directed violence (e.g., “Did anyone in your home ever intentionally hurt or threaten to hurt a pet?”) may help identify individuals whose developmental histories involve exposure to both interpersonal violence and animal cruelty (47, 49). Disclosure of both interpersonal violence and animal cruelty exposure indicates that a suicide risk assessment is warranted, especially in the presence of elevated psychological distress and limited social support.

It may also be beneficial to explicitly address experiences of animal-related trauma in trauma-focused treatments for survivors of childhood violence. Many trauma-informed treatment modalities, including trauma-focused cognitive behavioral therapy and EMDR (eye movement desensitization and reprocessing), allow for flexible targeting of traumatic memories and associated cognitions (85). Therefore, incorporating narratives about witnessing harm to animals, the grief associated with losing a pet to violence, and the guilt or shame of being unable to protect them could be of value. Such work could be especially relevant in suicide-focused interventions, where themes of self-blame, perceived burdensomeness, and moral injury often emerge (69, 86, 87).

Our findings also support the need for stronger collaboration between mental health services, domestic violence programs, and animal welfare and veterinary sectors (88). Professionals in animal-related fields may encounter individuals living in or fleeing violent homes where pets are also victimized or used as tools of coercive control (89). Training these providers to recognize the potential mental health risk implications of such disclosures, and establishing clear pathways for referral and cross-reporting (where legally permissible and ethically appropriate), could facilitate earlier identification and intervention for people at risk (90). Conversely, domestic violence and mental health services should consider animal safety when working with individuals exposed to co-occurring interpersonal violence and animal cruelty (91, 92).

### 4.7 Strengths and limitations

This study has several strengths. We used a person-centered latent class approach to characterize heterogeneity in threat-based adversity, allowing us to move beyond cumulative ACE scores and examine a specific pattern of co-occurring exposure to interpersonal violence and animal cruelty. The sample was large and reasonably diverse with respect to age, race/ethnicity, gender, and sexual orientation, increasing the relevance of the findings for community populations. We assessed multiple indicators of suicidality (i.e., two complementary measures of suicidal ideation and a lifetime suicide attempt item) providing a fuller representation of suicide risk than a single metric. Analytically, the use of the BCH method allowed us to adjust for classification error without altering the latent class structure, and models adjusted for overall psychological distress, social support, and key sociodemographic covariates, offering theoretically grounded tests of associations between adversity profiles and suicidality.

Several limitations should also be noted. First, the cross-sectional design and retrospective assessment of childhood experiences preclude causal inference. Although childhood adversity generally precedes adult suicidality, our measure of suicide attempts did not specify timing, meaning that some participants may have attempted suicide before experiencing interpersonal or animal-directed violence before the age of 18. Additionally, recall of childhood adversity may be influenced by current mental health or life circumstances, and we cannot determine the temporal ordering among psychological distress, social support, and suicidality. Psychological distress may represent an intermediate process through which childhood adversity influences suicidality; accordingly, adjusting for distress in cross-sectional models likely yields conservative estimates of the associations between adversity profiles and suicidality.

Second, our measurement of adversity was constrained. Threat-based ACE domains and childhood exposure to animal cruelty were dichotomized (any vs. no exposure), which obscures variation in severity, frequency, and chronicity. As noted in McDonald et al. (32), this approach likely elevates prevalence estimates and limits comparability with population-based ACE studies. Additionally, several forms of adversity were not assessed, including community violence, which is considered a threat-based ACE in dimensional models. Further, childhood exposure to animal cruelty was measured with only two items that did not capture severity, perpetrator identity, timing, or emotional closeness to the pet. Emotional attachment to the animal may shape the psychological impact of witnessing or hearing about pet-directed harm and could help explain heterogeneity in outcomes within exposure groups, as these features of violence exposure are known to influence developmental outcomes (93). Additionally, history of suicide attempt was measured with only a single BSS item, with no information on intent, lethality, or recency. Together, these measurement limitations likely attenuate effect sizes and limit the precision with which we can understand the relationship between different types of childhood adversity and suicidality.

Third, all data were collected via self-report in a single online survey, which introduces the possibility of shared method variance and socially desirable responding. Although anonymous online surveys may facilitate disclosure of sensitive experiences, they may also shape response patterns in unpredictable ways. Clinically verified diagnoses and multi-informant reports of adversity and support would help address these concerns in future research.

Fourth, although the sample was diverse, participants were recruited through Prolific and were not fully representative of the general population or of clinical samples. Participants were predominantly from the U.S., White/non-Hispanic, and overwhelmingly pet-owning in childhood and adulthood. Because participants self-selected into the study through an online platform until the target sample size was reached, a response rate could not be calculated, and individuals with interest in mental health or lived experience of suicidality may have been more likely to participate. Individuals without internet access or those less likely to participate in online research may be underrepresented. As described in McDonald et al. (32), these factors, and the way we measured ACEs (using multiple detailed items), likely contribute to elevated ACE prevalence and may limit generalizability to other cultural, socioeconomic, or clinical contexts.

Fifth, we did not assess several constructs that may mediate or confound associations between adversity profiles and suicidality, including substance use disorders, emotion regulation difficulties, or related mental health diagnoses (94–96). We also lacked direct measures of structural and contextual factors (e.g., neighborhood violence, discrimination, and poverty) that are intertwined with both adversity exposure and suicide risk. In addition, some racial and ethnic identities were grouped into broad categories to ensure sufficient statistical power; this approach is not best practice and may obscure heterogeneity in adversity exposure, risk and protective factors, and suicidality across specific subgroups (97).

Finally, the latent classes were developed in prior work with the same dataset. Although the classes were theoretically interpretable and consistent with person-centered studies of adversity, LCA is inherently sample-dependent. Class prevalence, structure, and associations with suicidality may differ in samples with different demographic compositions or adversity distributions. Replication in independent datasets is therefore needed to validate these profiles and to determine whether similar patterns emerge in other populations.

### 4.8 Future directions

Future research should improved the measurement of exposure to animal-directed violence in childhood. This includes assessing the timing, chronicity, and severity of exposure to animal cruelty; differentiating between direct victimization, witnessing, and being coerced to harm animals; and capturing the child’s emotional connection to the animals involved. Longitudinal studies following individuals from childhood into adulthood are needed to clarify how co-occurring human- and animal-directed violence shapes trajectories of mental health and suicidality over time, and to disentangle mediating pathways involving moral injury, attachment disruption, emotion regulation difficulties, and acquired capability for suicide. There is also a need to test whether explicitly addressing animal-related trauma and moral injury within existing trauma-focused and suicide-focused interventions improves outcomes for survivors of such experiences. Research should incorporate measures of structural and contextual factors (e.g., discrimination, housing instability, and neighborhood violence) to better understand racial/ethnic, gender, and sexual orientation disparities in both adversity exposure and suicidality. Finally, future work should replicate and extend these findings in clinical, forensic, inpatient, and non-U.S. samples, and should inform policies and practices in sectors where animal-related and human violence intersect, including veterinary medicine, animal shelters, and domestic violence services.

### 4.9 Conclusion

In summary, this study indicates that childhood exposure to co-occurring interpersonal violence and animal cruelty is associated with more intense suicidal thoughts and a greater likelihood of suicide attempt than either low adversity or exposure to interpersonal violence alone, even after accounting for psychological distress, social support, and sociodemographic factors. Sexual and gender minority and Black adults were more likely to report this high-risk adversity profile. These findings highlight childhood exposure to animal-directed violence as a clinically meaningful, yet frequently overlooked, component of threat-based adversity and suggest that its presence may mark families characterized by particularly severe and pervasive violence. Incorporating assessment of animal cruelty into trauma and suicide risk evaluations, and fostering collaboration across mental health, domestic violence, and animal welfare/health systems, may improve identification and support for individuals at elevated risk. Recognizing and addressing this form of adversity offers a concrete avenue for strengthening suicide prevention efforts and better serving survivors of complex violence.

## Data Availability

Data may be available upon request. Requests to access the datasets should be directed to shelby.e.mcdonald@colostate.edu.
